# *Aquilaria sinensis* essential oil inhibits biofilm formation and virulence of *Staphylococcus aureus*

**DOI:** 10.3389/fmicb.2025.1697407

**Published:** 2025-12-01

**Authors:** Fang Wang, Zhi-Wen Ding, Ying-Jie Wang, Kai-Zhong Xu, Owias Iqbal Dar, Min Wang, Sivasamy Sethupathy, Yang-Yang Liu, Shi Tang

**Affiliations:** 1 Key Laboratory of Tropical Biological Resources of Ministry of Education, School of Pharmaceutical Sciences, Hainan University, Haikou, China; 2 Key Laboratory of Ministry of Education for Advanced Materials in Tropical Island Resources, Department of Chemistry and Chemical Engineering, Hainan University, Haikou, China; 3 Department of Pharmacy, Hainan General Hospital (Hainan Affiliated Hospital of Hainan Medical University), Hainan, China; 4 Hainan Provincial Key Laboratory of Resources Conservation and Development of Southern Medicine & International Joint Research Center for Quality of Traditional Chinese Medicine, Hainan Branch of the Institute of Medicinal Plant Development, Chinese Academy of Medical Sciences and Peking Union Medical College, Haikou, China

**Keywords:** quorum sensing inhibitors, *Staphylococcus aureus*, *Aquilaria sinensis* essential oil, biofilms, virulent factors

## Abstract

*Staphylococcus aureus* is a major foodborne pathogen characterized by strong virulence and biofilm-forming ability, posing a persistent threat to food safety. *Aquilaria sinensis* (Lour.) Gilg, a medicinal and edible plant found in tropical regions such as Hainan, has rarely been investigated for its quorum-sensing inhibitory (QSI) potential. In this study, four types of *A. sinensis* essential oils were systematically evaluated for their ability to inhibit quorum sensing and related virulence in *S. aureus*. Among them, the Tong-Ti-Xiang type supercritical essential oil (TTC) exhibited the strongest activity. At a sub-inhibitory concentration of 125 μg/mL (1/4 MIC), TTC reduced biofilm formation by up to 78% without affecting bacterial growth. Confocal laser scanning Microscopy and scanning electron microscopy analyses further confirmed that TTC compromised the density and structural integrity of the biofilms. Moreover, TTC suppressed α-hemolysin activity and significantly downregulated quorum-sensing-regulated genes involved in biofilm formation and adhesion, including *icaA*, *icaD*, *clfA*, *clfB*, and *agrA*. Gas chromatography mass spectrometry analysis revealed that TTC is rich in chromone derivatives, among which 2-phenethyl-4H-chromen-4-one showed strong binding affinity toward the adhesion-associated IcaA protein and the QS-associated AgrA protein in molecular docking analysis, suggesting it as a key active compound responsible for the observed QSI effect. Overall, these findings highlight TTC as a promising natural anti-biofilm and anti-virulence agent for controlling foodborne pathogens.

## 1 Introduction

*Staphylococcus aureus*, a facultative anaerobic gram-positive bacterium, is recognized as a major foodborne pathogen of particular concern to the food industry. It has the potential to contaminate protein-rich foods, including dairy products, meat, eggs, and seafood, thereby posing significant threats to food safety (Kang et al., 2019). This pathogen produces various heat-stable enterotoxins that cause staphylococcal food poisoning (SFP), characterized by acute gastrointestinal symptoms (Aydin et al., 2011). Notably, *S. aureus* can adhere to food-contact surfaces and form biofilms, producing extracellular matrices which is composed of polysaccharides, proteins, and eDNA (Elafify et al., 2024). Biofilm formation enhances bacterial resistance to environmental stresses (e.g., temperature and pH fluctuations), disinfectants, and antibiotics, complicating sanitation procedures and leading to persistent contamination in food processing environments (Galié et al., 2018).

In the food industry, the application of chemical preservatives is widespread, primarily aimed at inhibiting microbial contamination and extending product shelf-life. However, prolonged use of synthetic preservatives may lead to issues such as enhanced bacterial resistance and potential toxic risks (Buckley et al., 2017). In recent years, plant essential oils have garnered significant attention due to their natural antimicrobial properties and low/non-toxic nature. These aromatic oily substances, extracted from fragrant plants, exhibit broad-spectrum antibacterial effects and represent a safer alternative to synthetic preservatives (Pandey et al., 2016). Research has demonstrated that certain essential oils show remarkable inhibitory effects against pathogens in high-protein, low-fat, or low-carbohydrate acidic food products, indicating promising application prospects (Gutierrez et al., 2008).

*Aquilaria sinensis* essential oil, derived from agarwood, is rich in various chromone derivatives, sesquiterpenes, and other bioactive compounds. Previous studies have demonstrated its antimicrobial, anti-inflammatory, and immunomodulatory properties (Wang S. et al., 2018). In recent years, several plant essential oils have been reported to interfere with bacterial quorum sensing (QS) systems. This interference reduces the expression of virulence factors and inhibits biofilm formation, exhibiting potential as quorum sensing inhibitors (QSIs) (Luciardi et al., 2020). However, whether *A. sinensis* essential oil possesses similar QSI activity against *S. aureus* remains unclear. Given its diverse composition, studying this essential oil could reveal new insights into its antimicrobial mechanisms. Unlike previous studies, mainly focused on common plant essential oils such as peppermint, lemongrass, or grapefruit, this study investigates the QSI potential of *A. sinensis* essential oils. In this study, we have chosen to explore two widely recognized *A. sinensis* essential oils—Agar-Wit (Tong-Ti-Xiang) and Burning-Chisel-Drilling (Huo-Lao-Xiang) were evaluated for their anti-biofilm and anti-virulence activities *in vitro* against *S. aureus*. Notably, the Tong-Ti-Xiang type supercritical essential oil (TTC) was identified as the most effective quorum sensing inhibitor. Our findings aim not only to contribute to the development of natural antimicrobial alternatives for food systems but also to illuminate non-antibiotic strategies for enhancing food safety, paving the way for a healthier future in food production and consumption.

## 2 Materials and methods

### 2.1 Bacterial strains, culture medium, and reagents

Four agarwood essential oils were kindly provided by Prof. Yangyang Liu from the Hainan Branch of the Institute of Medicinal Plant Development, Chinese Academy of Medical Sciences and Peking Union Medical College. The plant materials originated from *Aquilaria sinensis* (Lour.) Gilg of the Thymelaeaceae family. Information regarding their extraction methods and sources is listed in Supplementary Table S1. Unless otherwise indicated, all chemicals used in this study were of analytical grade. The *Staphylococcus aureus* reference strain ATCC 25923 was obtained from Huankai Microbial Science & Technology Co., Ltd. (Guangdong, China).

### 2.2 QSI activity of *A. sinensis* essential oil

*Staphylococcus aureus* was first cultured in tryptic soy broth (TSB) within shaking flasks at 28 °C, 180 rpm, for approximately 17 h. The bacterial suspension was then standardized to an optical density of 0.05 at 620 nm (OD₆₂₀). For the plate assay, 1 mL of this suspension was incorporated into 100 mL of molten TSB agar, mixed thoroughly, and poured into Petri dishes. After the agar solidified, agarwood essential oil was accurately weighed and dissolved in DMSO to prepare a stock solution (100 mg/mL) for preliminary screening. Sterile paper disks on the agar surface were loaded with 5 μL of each test solution, while disks containing the same volume of DMSO served as negative controls. The plates were incubated without shaking at 28 °C for 24 h before assessment (Zhou et al., 2019).

### 2.3 MICs and growth curves determination of *A. sinensis* essential oils

In a 96-well microtiter plate (Corning, New York, United States), 2 μL of the prepared essential oil stock was added into 200 μL of bacterial suspension, adjusted to approximately 1.5 × 10^5^–10^6^ CFU/mL. The inoculated plates were incubated at 37 °C for 24 h, after which the minimum inhibitory concentrations (MICs) were determined (Lu et al., 2022).

A bacterial suspension prepared as previously described (200 μL per well) was dispensed into a 96-well plate. Varying concentrations of *A. sinensis* essential oil (125 μg/mL and 62.5 μg/mL) were then introduced and thoroughly mixed. The cultures were incubated at 37 °C under agitation (180 rpm). To assess the influence of the essential oil at sub-inhibitory levels (125 μg/mL and 62.5 μg/mL), bacterial growth was monitored at OD₆₂₀ using a microplate reader at 3.5 h intervals. Growth dynamics were subsequently analyzed and visualized with GraphPad Prism 9.0 (Huband et al., 2020).

### 2.4 Assessment of biofilm inhibition by *A. sinensis* essential oils against *S. aureus*

*Staphylococcus aureus* was cultured overnight in 5 mL TSB medium at 37 °C with shaking (200 rpm) for 12 h. The resulting culture was adjusted with sterile TSB to approximately 10^5^ CFU/mL. Aliquots of 100 μL were transferred into the wells of a 96-well microplate. Wells containing sterile DMSO served as negative controls. The plates were maintained without shaking at 37 °C for 24 h to allow biofilm establishment. After incubation, residual medium was removed, and each well was gently rinsed three times with 200 μL sterile PBS (10 mM, pH 7.2) to eliminate unattached cells. Biofilms were fixed using methanol (1:1 volume) for 15 min, followed by staining with 0.05% crystal violet for 10 min. Excess dye was removed by washing with PBS, and the retained stain was eluted with 200 μL of 95% ethanol at 37 °C for 30 min. The absorbance of the extracted dye was recorded at 570 nm (Xu et al., 2024).

### 2.5 GC/MS measurement of TTC and QS suppression by the major components

The GC oven program was initiated at 50 °C for 1 min, followed by a gradual rise to 250 °C at a rate of 5 °C/min, and maintained at 250 °C for an additional 5 min. The column pressure was kept constant at 50 kPa, while the carrier gas was delivered at 1.2 mL/min with a split ratio of 1:20. Mass spectrometric detection was carried out in electron impact mode (70 eV ionization energy). The ion source was maintained at 230 °C, and data were collected over a mass range of 20–500 m/z. Compound identification was achieved by matching the acquired spectra against the NIST 14 reference library, and the relative proportions of individual constituents in TTC were determined using peak area normalization (Jiang et al., 2024). For the initial evaluation of quorum sensing inhibitory activity, three representative constituents—2-phenethyl-4H-chromen-4-one,6-methoxy-2-phenethyl-4H-chromen-4-one, and 1,2,4-triethylbenzene—were chosen. Each compound was precisely weighed and dissolved in DMSO to yield stock solutions at 100 mg/mL. The subsequent activity assessment was carried out following the procedure outlined in Section 2.2.

### 2.6 Confocal laser scanning microscopy (CLSM) measurement

Biofilms were established and rinsed according to the procedure described earlier, followed by staining with acridine orange solution (0.1 mg/mL) for 15 min. After removing residual dye with PBS buffer (pH 7.2, 10 mM), the samples were observed using a confocal laser scanning microscope (CLSM, Leica TCS SP8, Wetzlar, Germany). Fluorescence was detected under excitation at 488 nm and emission in the range of 501–545 nm. For each group, five randomly selected fields were imaged and analyzed (Ding et al., 2025).

### 2.7 Scanning electron microscopy (SEM) measurement

The cultured biofilms were washed with PBS to remove non-adherent cells from the glass slides, then fixed with 2.5% glutaraldehyde (Aladdin Biochemical Technology Co., Ltd., China). After fixation, the biofilms were dehydrated through a graded ethanol series (50, 70, 80, 90, and 100%), followed by freeze-drying and gold sputter-coating. SEM imaging was performed using a JSM-6360 instrument (JEOL, Tokyo, Japan) (Sutipornpalangkul et al., 2021).

### 2.8 α-hemolysin activity assay

An overnight culture of *S. aureus* was inoculated into fresh TSB medium at 1% (v/v), followed by the addition of agarwood essential oil at final concentrations of 125 μg/mL and 62.5 μg/mL. DMSO at the same volume served as the negative control. The inoculated medium (1 mL/well) was distributed into 24-well plates with three parallel wells for each condition and incubated at 37 °C for 24 h. After cultivation, the bacterial suspensions were centrifuged at 10,000 rpm for 10 min at 4 °C to obtain cell-free supernatants. Subsequently, 100 μL of the supernatant was combined with 900 μL of sterile 5% sheep blood (prepared in PBS, pH 7.2). The mixtures were incubated at 37 °C for 1 h and then centrifuged at 3,000 rpm for 10 min. Hemolytic activity was determined by measuring the absorbance of the supernatant at 530 nm (Wan et al., 2022).

### 2.9 Quantitative real-time polymerase chain reaction (RT-qPCR)

*S. aureus* was grown in TSB medium supplemented with 125 μg/mL TTC or an equal volume of DMSO as the control at 37 °C with shaking at 180 rpm for 24 h. The bacterial pellets were collected by centrifugation at 8,000 × g for 15 min at 4 °C. Total RNA was isolated using the UNIQ-10 Column Trizol Total RNA Isolation Kit (Sangon Biotech, China), and reverse transcription was carried out with a commercial kit (Biosharp, China) to obtain cDNA. Quantitative real-time PCR (qRT-PCR) was conducted with BeyoFast™ SYBR Green qPCR Mix (Beyotime, China) on a Bio-Rad CFX Connect platform (Bio-Rad, United States). The primers applied in this work are listed in Supplementary Table S2. The 16S rRNA gene was used as the reference control, and the relative transcription levels of target genes were analyzed using the 2^−ΔΔCt^ method (Sun et al., 2021).

### 2.10 Molecular docking analysis

Protein structure was retrieved from the UniProt database^1^ by querying target genes. The 3D structure of 2-phenethyl-4H-chromen-4-one in SDF format was downloaded from the PubChem database^2^. The SDF file was converted to PDB format using Open Babel 3.1.1. Molecular docking simulations were performed using AutoDockTools 1.5.6 and AutoDock software. The 2D interaction diagrams were generated with LigPlus and electrostatic potential visualization, while 3D structures were rendered using PyMOL 2.3.0 (Wang et al., 2024).

### 2.11 Statistical analysis

All assays were conducted in triplicate or more. Statistical analyses and graph generation were carried out using GraphPad Prism 9.0 (San Diego, CA, United States). The notation ns represents no significant difference between the agarwood essential oil treatment and the DMSO control. Symbols *, **, and *** correspond to statistical significance levels of *p* < 0.05, *p* < 0.01, and *p* < 0.001, respectively, as determined by one-way ANOVA in comparison with the DMSO group.

## 3 Results

### 3.1 MICs, quorum sensing inhibitory zones, and growth curve assays

**Figure 1:**
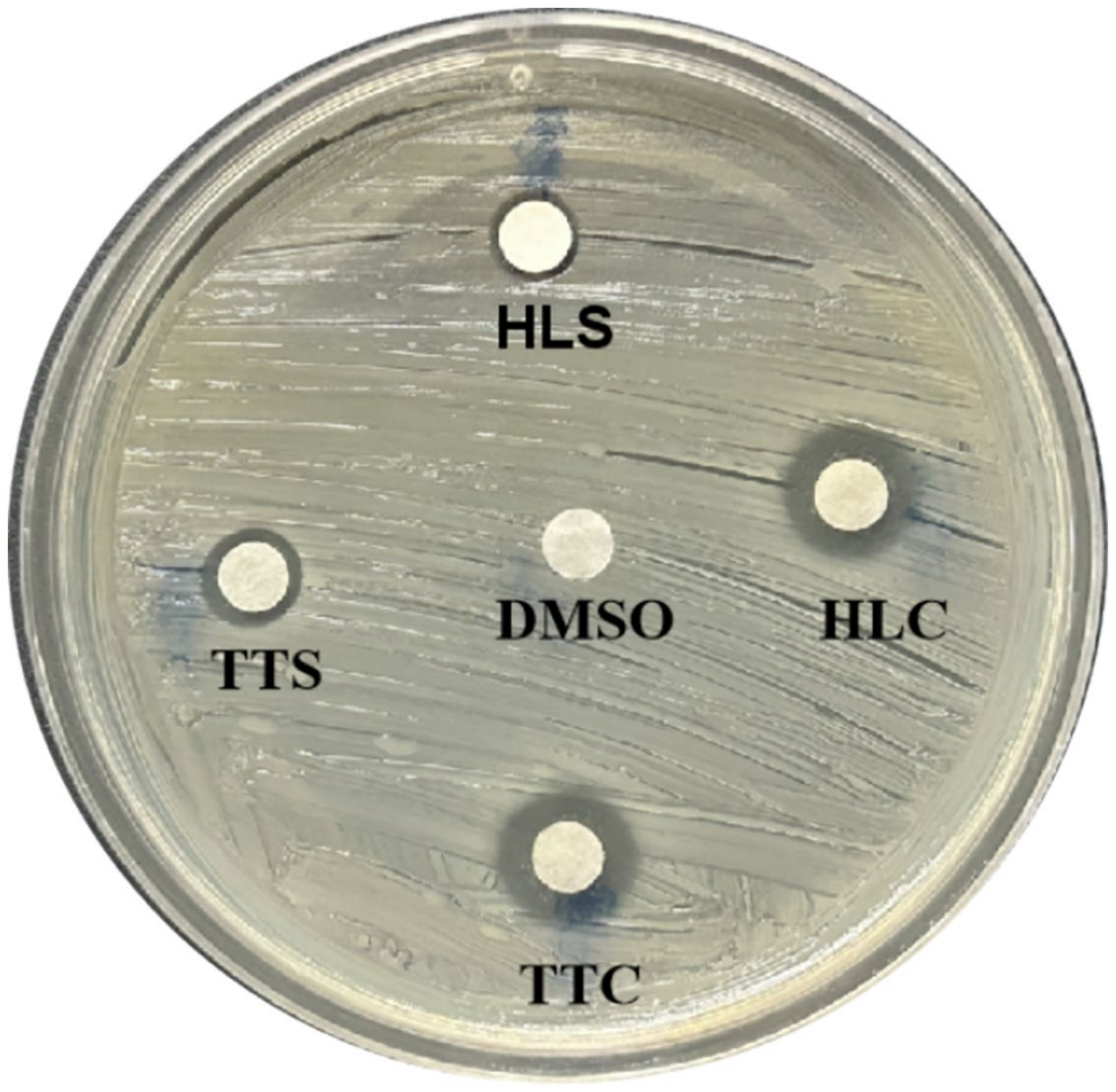
Quorum sensing (QS) inhibitory zones of four agarwood essential oils (TTC, TTS, HLS, HLC) on *S. aureus*. All test concentrations were 100 mg/mL. Three replicate plate experiments were performed.

The MICs of four agarwood essential oils against *S. aureus* were, respectively (Table 1). As shown in Figure 1, TTC, TTS, HLC, and HLS all exhibited QSI activity, among which TTC showed the best QSI effects (Figure 1).

**Table 1:** MICs of four agarwood essential oils against *S. aureus.*

Sample	MIC (μg/mL)
TTC	500
TTS	250
HLS	250
HLC	500

**Figure 2:**
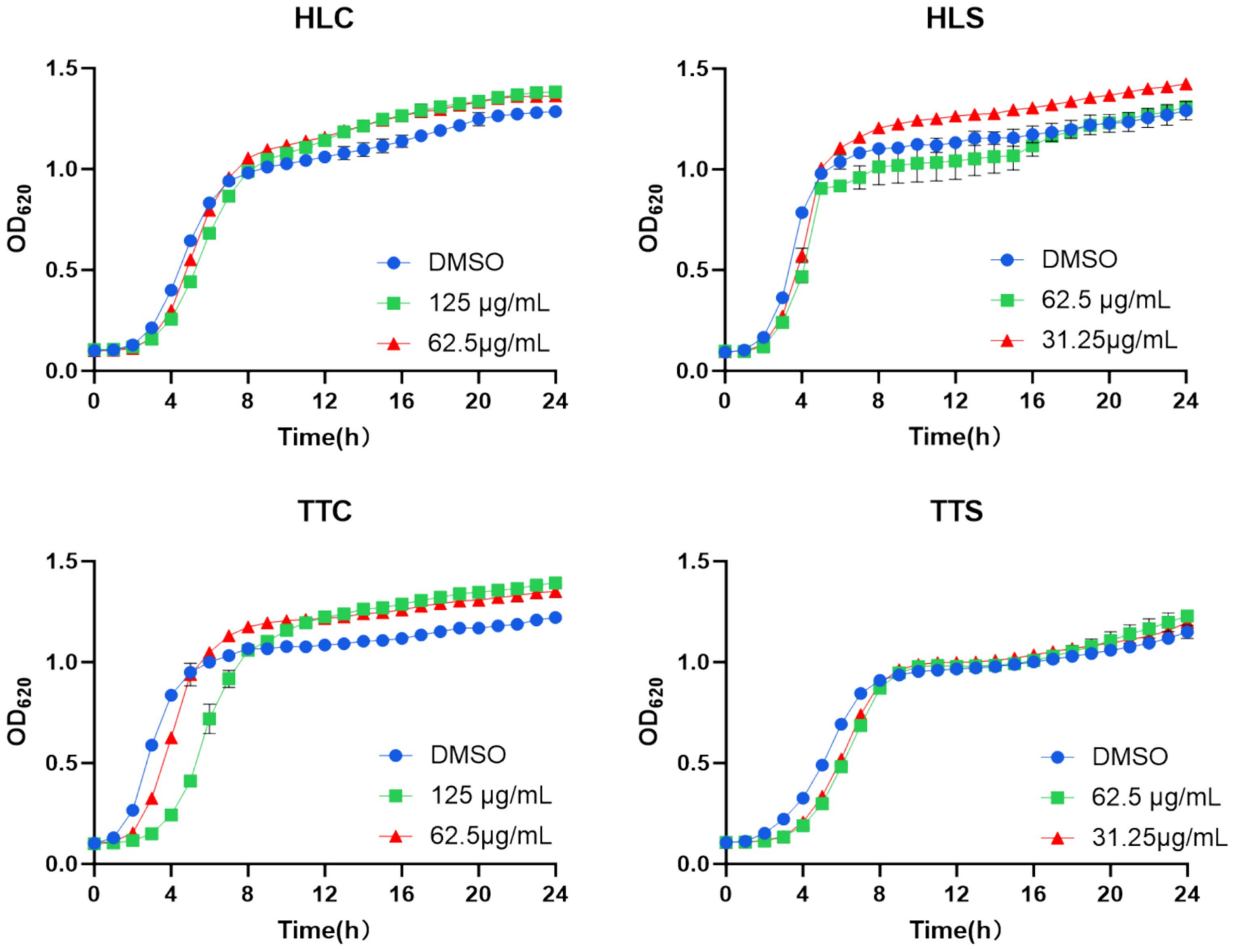
Effects of four agarwood essential oils on the 24-h growth of *S. aureus. D*ata are presented as mean ± standard deviation (*n* = 3).

The QSI are chemical substances that interfere with bacterial biofilm formation and virulence factor synthesis by affecting the QS system without impacting bacterial growth (Eickhoff and Bassler, 2018). Therefore, this study examined whether the four selected agarwood essential oils at sub-MIC concentrations (125 μg/mL and 62.5 μg/mL) affected the growth of *S. aureus*, with results shown in Figure 2. The 24-h growth of *S. aureus* was not significantly inhibited by any of the four essential oils at 125 μg/mL concentration. Consequently, the 125 μg/mL and 62.5 μg/mL concentrations of all four essential oils were selected to evaluate their QSI activity against *S. aureus*, to identify the essential oil exhibiting the most potent QSI activity.

### 3.2 Inhibitory effects of four agarwood essential oils on biofilm formation

**Figure 3:**
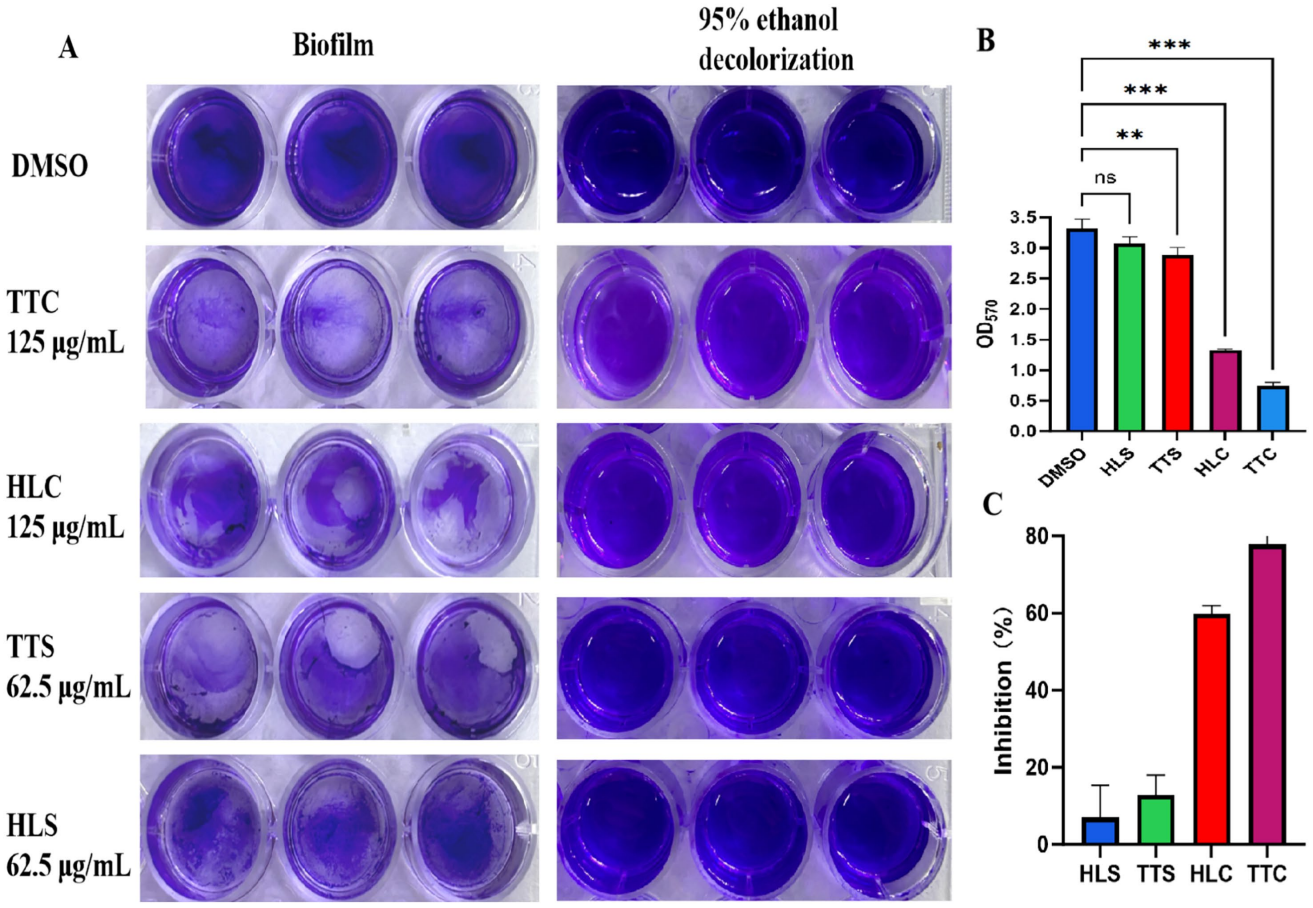
**(A)** Visualization of biofilm growth on polyethylene material. **(B)** Quantitative analysis of biofilm. **(C)** Inhibition rate of compounds on biofilm. Data are presented as mean ± standard deviation (*n* = 3), ns indicates no statistical significance, ** indicated the very significant differences (*p* < 0.01) between TTC group and DMSO group by one-way ANOVA. *** indicated the extremely significant differences (*p* < 0.001) compared to the DMSO group by one-way ANOVA.

Biofilm formation capacity of *S. aureus* treated with four agarwood essential oils was evaluated. As shown in Figure 3, after 125 μg/mL treatment, TTC and HLC significantly inhibited the formation of biofilms on polyethylene material (Figure 3A), with inhibition rates was 78 and 59%, respectively, (Figure 3C), showing statistically significant differences compared to the control group (*p* < 0.001). The TTS group only inhibited slightly, and the differences were not significant.

### 3.3 GC–MS analysis of Tong-Tian-Xiang essential oil by supercritical fluid extract of CO_2_ (SFE-CO_2_)

By using gas chromatography–mass spectrometry (GC–MS) technology, a total of 10 major compounds were identified (Table 2). The results showed that the predominant constituents of the essential oil were 2-phenethyl-4H-chromen-4-one (7.33%), 2-(4-methoxyphenethyl)-4H-chromen-4-one (3.93%), and 6-methoxy-2-phenethyl-4H-chromen-4-one (3.01%). In addition to these chromone derivatives, the oil also contained sesquiterpenoids such as longifolene (0.61%), agarospirol (0.40%), and 2-((2S,4aR)-4a,8-dimethyl-1,2,3,4,4a,5,6,7-octahydronaphthalen-2-yl)propan-2-ol (0.32%), together with oxygenated sesquiterpenes like 7-(2-hydroxypropan-2-yl)-1,4a-dimethyldecahydronaphthalen-1-ol (0.60%). Furthermore, fatty acid components such as oleic acid (0.42%) were also detected. These findings indicate that, besides chromone-type aromatics, the essential oil obtained by supercritical CO₂ extraction also comprises sesquiterpenoids, fatty acids, and other oxygenated derivatives, highlighting the chemical diversity and complexity of agarwood essential oil.

**Table 2:** Major chemical constituents of TTC supercritical CO₂-extracted agarwood oil.

No.	Compounds	MF.	Rt	Relative area percentage (%)
1	2-phenethyl-4H-chromen-4-one	C_17_H_14_O_2_	37.17	7.33
2	2-(4-Methoxyphenethyl)-4H-chromen-4-one	C_18_H_16_O_3_	41.35	3.93
3	6-methoxy-2-phenethyl-4H-chromen-4-one	C_18_H_16_O_3_	41.24	3.01
4	2(3H)-naphthalenone,4,4a,5,6,7,8-hexahydro-4a,5-dimethyl-3-(1-methylethylidene)-, (4ar-cis)-	C_15_H_22_O	27.41	1.20
5	6,7-dimethoxy-2-phenethyl-4H-chromen-4-one	C_19_H_18_O_4_	45.11	1.05
6	Longifolene	C₁₅H₂₄	23.94	0.61
7	7-(2-Hydroxypropan-2-yl)-1,4a-dimethyldecahydronaphthalen-1-ol	C₁₅H₂₆O_2_	26.73	0.60
8	Oleic Acid	C₁₈H₃₄O₂	33.68	0.42
9	Agarospirol	C₁₅H₂₆O	23.74	0.40
10	2-((2S,4aR)-4a,8-Dimethyl-1,2,3,4,4a,5,6,7-octahydronaphthalen-2-yl)propan-2-ol	C₁₅H₂₆O	23.40	0.32

**Figure 4:**
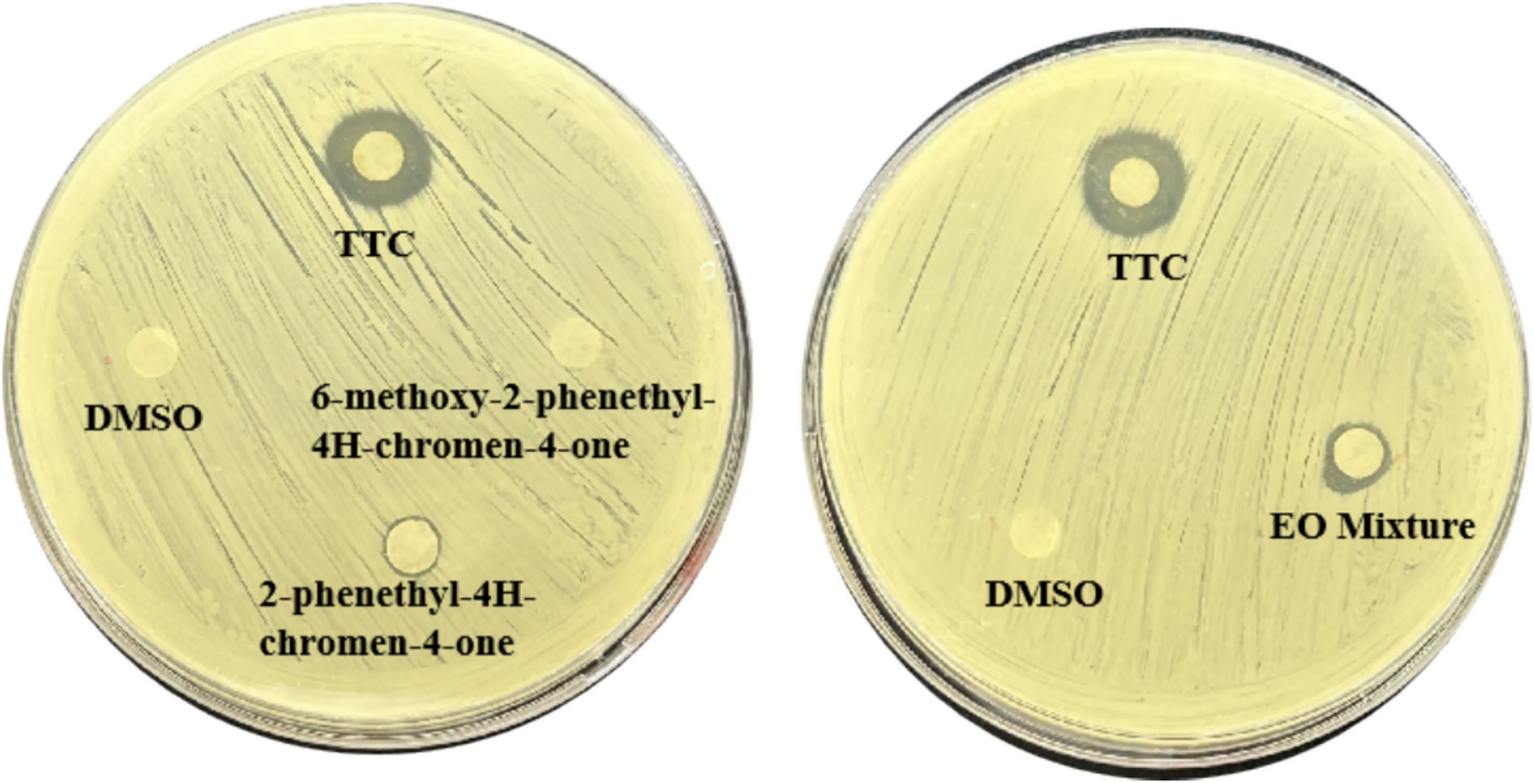
Compare the QSI activity of TTC and its main components against *S. aureus.*

To further clarify the contribution of the major components of TTC to its quorum sensing (QS) inhibitory activity, three dominant constituents— 2-Phenethyl-4H-chromen-4-one and 6-Methoxy-2-phenethyl-4H-chromen-4-one were individually evaluated using the agar diffusion preliminary screening assay. As shown in Figure 4, 2-Phenethyl-4H-chromen-4-one exhibited a stronger QS inhibitory activity compared with the other components; however, its activity remained lower than that of the complete TTC essential oil. In addition, the two major components were mixed in the same proportion as detected in TTC and assessed for QS inhibitory activity. As illustrated in Figure 2, the mixed compounds displayed a higher inhibitory activity than 2-Phenethyl-4H-chromen-4-one alone, but the activity was still weaker than that of TTC. These results suggest that although 2-Phenethyl-4H-chromen-4-one contributes substantially to the QS inhibitory effect of TTC, the superior activity of TTC may be attributed to the synergistic interactions among multiple constituents.

### 3.4 CLSM analysis

**Figure 5:**
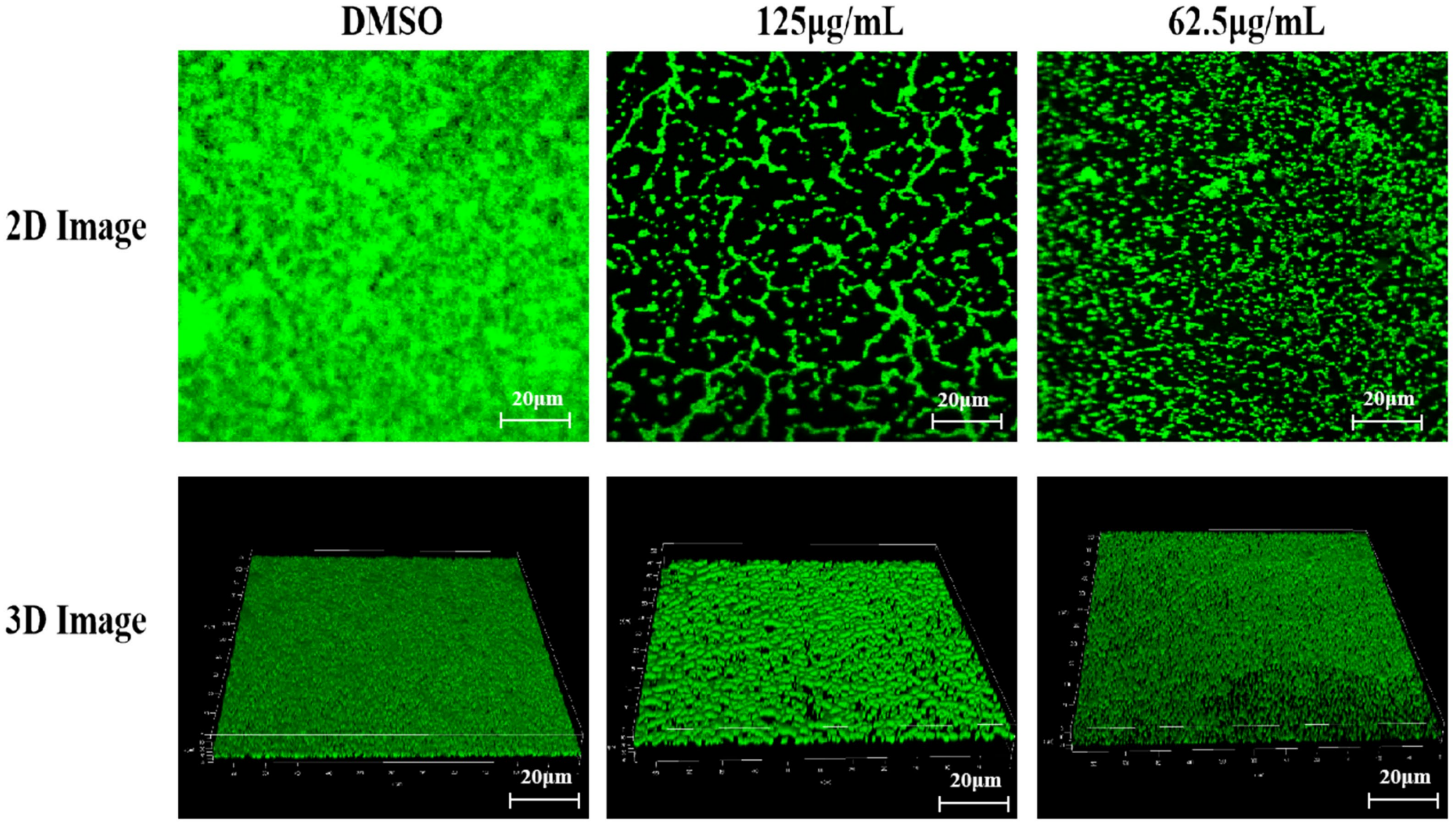
2D and 3D CLSM images of *S. aureus* treated with DMSO, 125 μg/mL TTC, and 62.5 μg/mL TTC (scale bar = 20 μm).

As shown in Figure 5A, the biofilm structure formed by bacteria in the DMSO-treated group was dense and interconnected. When treated with 125 μg/mL, the biofilm structure became loose and fragmented (Figure 5B). Furthermore, TTC exhibited increasingly significant inhibition of biofilms with increasing concentration, showing concentration-dependent inhibition. The fluorescence values (excitation wavelength 488 nm, emission wavelength 501–545 nm) of biofilms treated with 125 μg/mL and 62.5 μg/mL TTC were 23.99 and 40.78% of the control group, respectively.

### 3.5 SEM analysis

**Figure 6:**
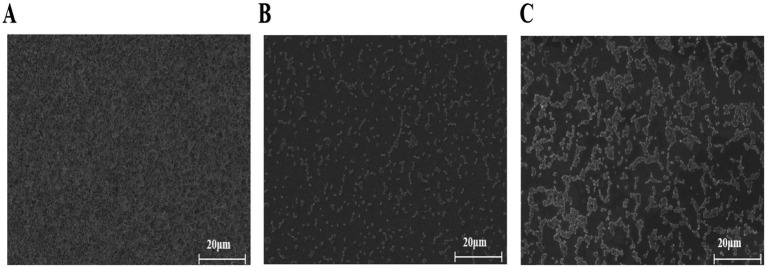
SEM images of *S. aureus* treated with **(A)** DMSO, **(B)** 125 μg/mL TTC, and **(C)** 62.5 μg/mL TTC (scale bar = 20 μm).

The SEM observations revealed notable alterations in biofilm morphology following TTC treatment (Figure 6). In the DMSO control group, the *S. aureus* biofilm densely covered the entire coverslip surface (Figure 5A). However, when exposed to 125 μg/mL or 62.5 μg/mL TTC, the biofilms displayed obvious structural disruption, with fewer bacterial cells and incomplete surface colonization (Figures 5B,C). These results suggest that TTC is capable of impairing biofilm development in *S. aureus* even at relatively low concentrations.

### 3.6 α-hemolysin activity assay

**Figure 7:**
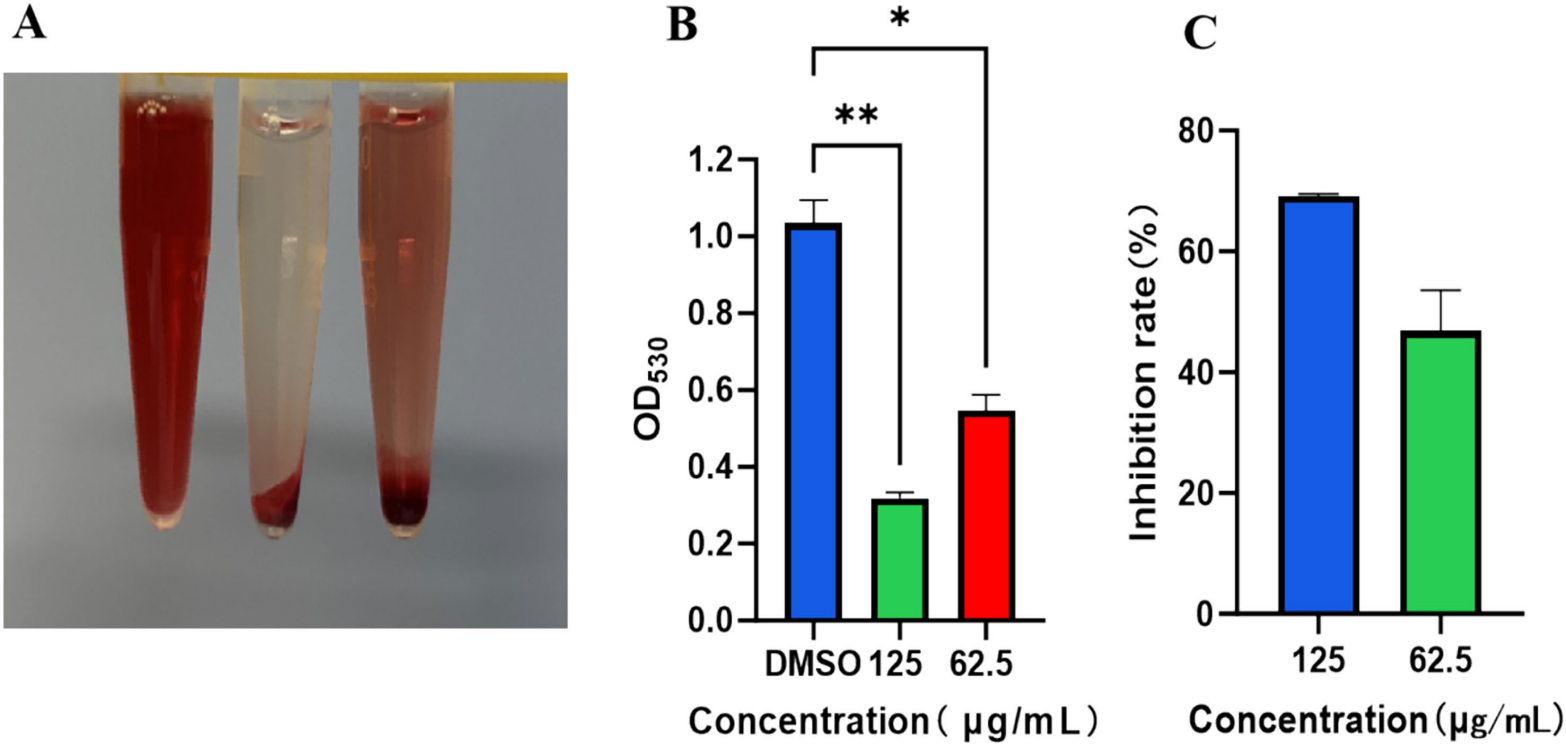
Inhibitory effect of TTC on α-hemolysin activity of *S. aureus*. **(A)** Representative images of erythrocyte lysis assay results. **(B)** Quantitative analysis of erythrocyte lysis degree. **(C)** Inhibition rate of hemolysis by TTC. Data are presented as mean ±SD (*n* = 3). * indicated the significant differences (*p* < 0.05) compared to the DMSO group by one-way ANOVA. ** indicated the very significant differences (*p* < 0.01) between TTC group and DMSO group by one-way ANOVA.

As shown in Figures 7A–C, significant erythrocyte lysis was observed in the DMSO-treated group, manifested by bright red supernatant. In contrast, TTC at concentrations of 125 μg/mL and 62.5 μg/mL effectively inhibited hemolysis, with near-complete suppression observed at 125 μg/mL. Statistical analysis revealed significant differences compared to the DMSO control group (**p < 0.01), demonstrating that TTC exhibits dose-dependent inhibition of α-hemolysin activity.

### 3.7 Effects of TTC on gene expression

**Figure 8:**
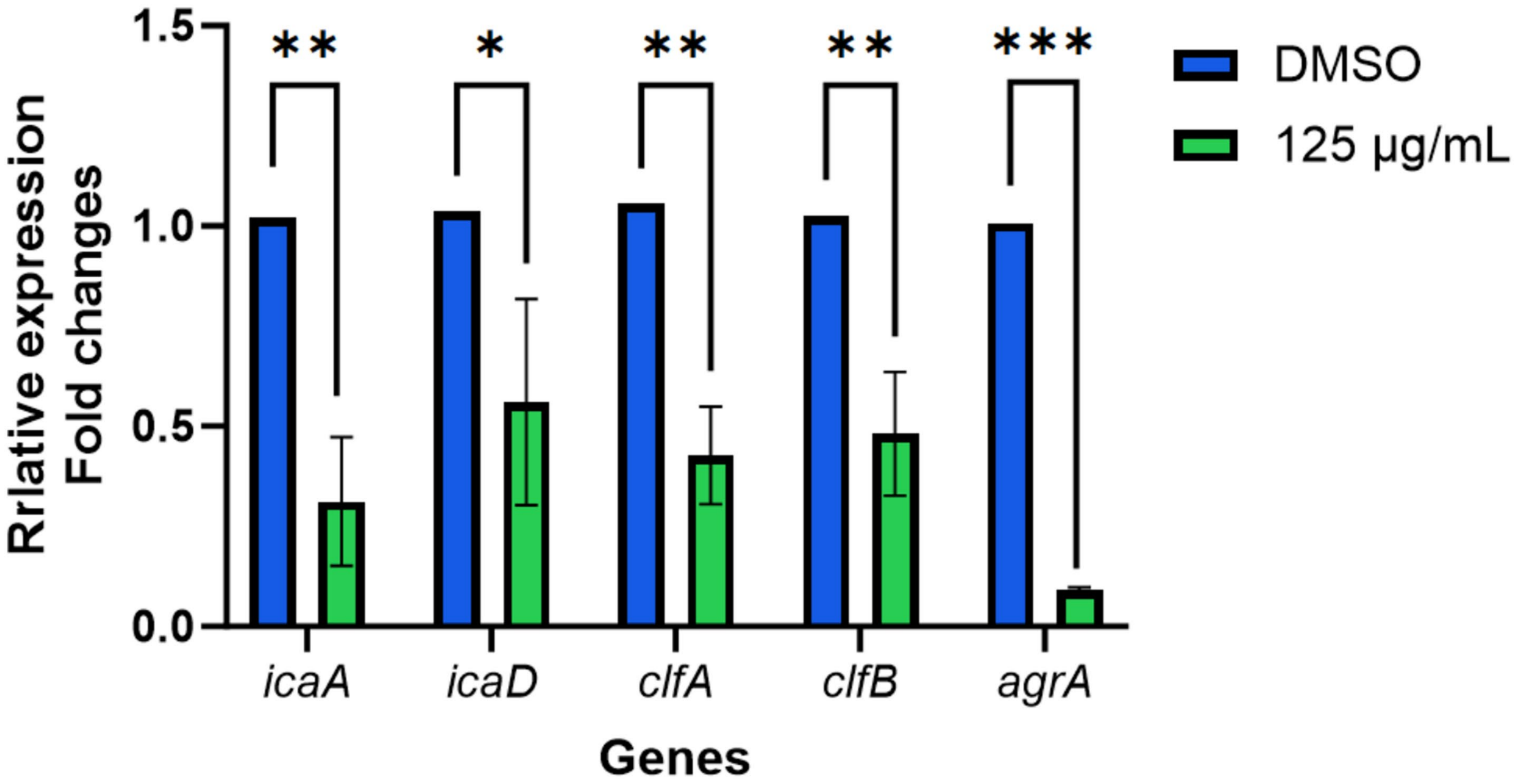
The effects of TTC on the genes expression of *S. aureus*. The relative expression of target genes was calculated using 2^−ΔΔCt^ method. Error bars represent the standard deviation (*n* = 3). * indicated the significant differences (*p* < 0.05) compared to the DMSO group by one-way ANOVA. ** indicated the very significant differences (*p* < 0.01) between TTC group and DMSO group by one-way ANOVA. *** indicated the extremely significant differences (*p* < 0.001) compared to the DMSO group by one-way ANOVA.

Using RT-qPCR analysis, we examined the transcriptional levels of key adhesion- and quorum-sensing–related genes in *S. aureus.* The results demonstrated that treatment with 125 μg/mL TTC led to a significant downregulation of multiple target genes (Figure 8). Specifically, the expression of *icaA* and *icaD* was markedly reduced, suggesting that TTC may inhibit the synthesis of polysaccharide intercellular adhesin (PIA), thereby weakening the bacterium’s ability to form biofilms. Likewise, the transcriptional levels of *clfA* and *clfB* were significantly decreased, indicating that TTC impairs the expression of clumping factor proteins and consequently diminishes bacterial adhesion to host proteins and extracellular matrices. In addition, the expression of *agrA* was notably suppressed, implying that TTC interferes with the QS regulatory system of *S. aureus*, which in turn affects the transcription and secretion of downstream virulence factors. Collectively, these findings suggest that TTC exerts a multi-target inhibitory effect on *S. aureus* by suppressing biofilm formation, adhesion capacity, and virulence regulation, highlighting its strong potential as an anti-pathogenic agent.

### 3.8 Molecular docking analysis

**Figure 9:**
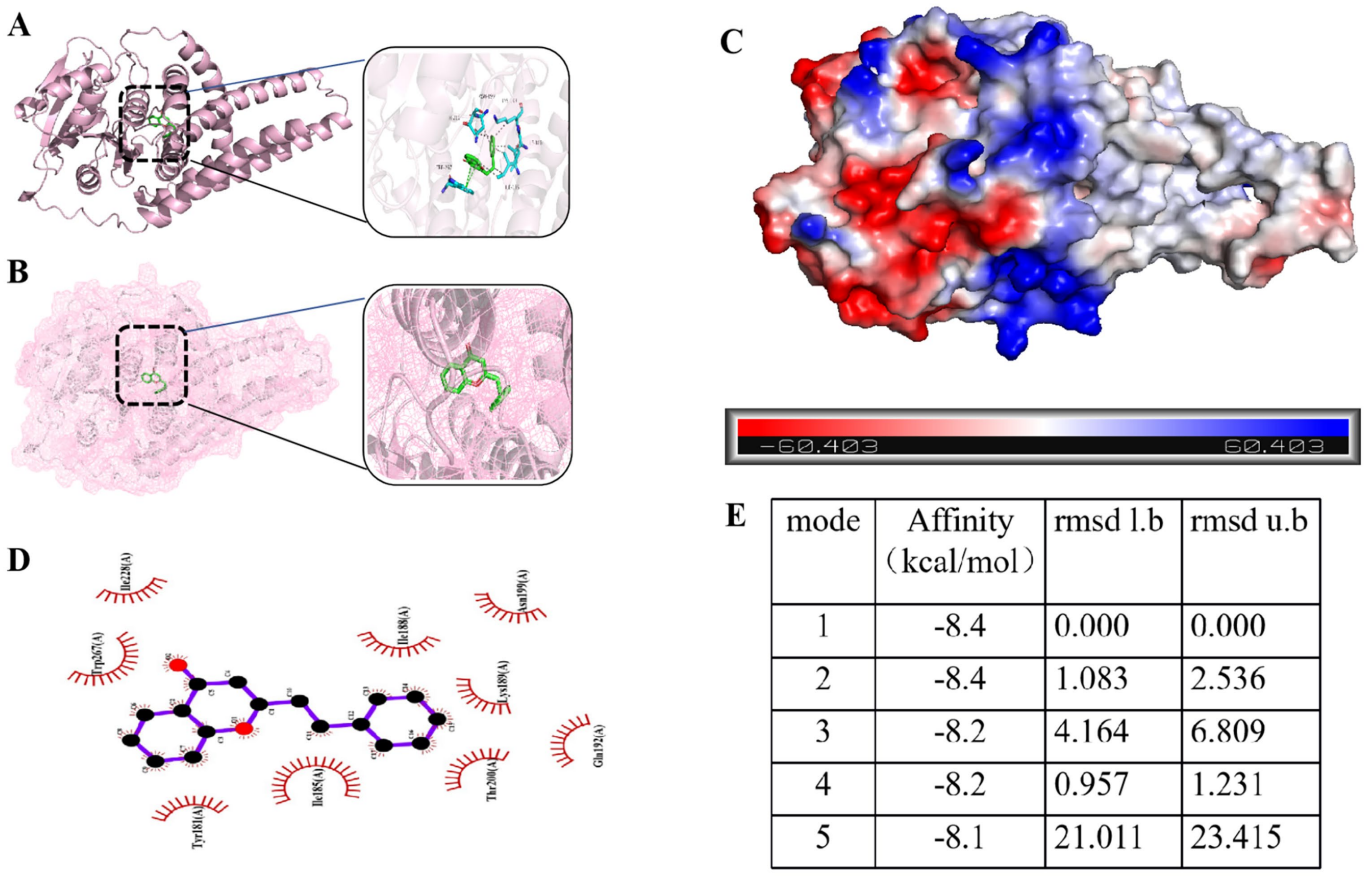
**(A)** Three-dimensional structure of 2-phenethyl-4H-chromen-4-one-*icaA* molecular docking. **(B)** 2-phenethyl-4H-chromen-4-one is located within the molecular pocket of the *icaA* protein. **(C)** Surface electrostatic forces of 2-phenethyl-4H-chromen-4-one-*icaA*. **(D)** Two-dimensional structure of 2-phenethyl-4H-chromen-4-one-*icaA*. **(E)** Lowest binding energy conformation of 2-phenethyl-4H-chromen-4-one molecular docking.

Molecular docking analysis was performed to investigate the interaction between 2-phenethyl-4H-chromen-4-one in TTC and the *icaA* protein. The docking results (Supplementary Figures S1–S3) showed that 2-phenethyl-4H-chromen-4-one could effectively bind to the active site of *icaA* protein with a minimum binding energy of −8.4 kcal/mol (Figures 9A,B,E). Further analysis revealed that this compound interacts with the *icaA* protein through various intermolecular forces, including electrostatic interactions, van der Waals forces, and hydrophobic effects (Figures 9C,D). These interactions enable 2-phenethyl-4H-chromen-4-one to form a stable complex with *icaA* protein, demonstrating strong binding affinity.

## 4 Discussion

*Staphylococcus aureus* is one of the most common and hazardous foodborne pathogens in the food industry, particularly prone to contaminating high-protein foods such as dairy products, meat, and egg products. *S. aureus* can produce various heat-stable enterotoxins that cause staphylococcal food poisoning (SFP), leading to acute gastrointestinal symptoms and significantly impacting public health (Kang et al., 2019). Additionally, *S. aureus* exhibits strong surface adhesion and biofilm formation capabilities, enabling persistent colonization on food processing equipment, packaging materials, and food surfaces. This makes it difficult to eliminate completely and thus poses a potential source of cross-contamination (Zhao et al., 2017). Recent studies have found that the QS systems in *S. aureus* play a key regulatory role in virulence factor secretion and biofilm formation. QSIs have emerged as a novel strategy to attenuate pathogenicity and recover antibiotic sensitivity, and have also become an important research direction for controlling foodborne pathogens (Vinodhini and Kavitha, 2024). Numerous plant essential oils have been reported to possess QSI activity (Szabó et al., 2010), but systematic studies on agarwood essential oils interfering with the QS system are still lacking. As a traditional medicinal and edible plant-derived volatile oil in Hainan, China, agarwood essential oils are rich in various chromone derivatives, various sesquiterpenes, and other bioactive compounds (Wang M. R. et al., 2018). Therefore, it is necessary to explore its potential anti-biofilm and anti-virulence to apply in food safety control.

In this study, TTC exhibits significant quorum-sensing inhibition activity at sub-MIC levels without affecting *S. aureus* growth (Figure 3). It markedly reduced biofilm formation by 78%, as confirmed by confocal laser scanning microscopy (CLSM) and scanning electron microscopy (SEM), which revealed that TTC-treated biofilms displayed loose and fragmented structures (Figures 5, 6). Previous work has studied the antimicrobial activity of agarwood essential oil primarily against *Bacillus subtilis* and *S. aureus* (Chen et al., 2011), while no reports have been made on its anti-biofilm and anti-virulence effects. In this work, agarwood essential oils were first investigated to effectively inhibit biofilm formation at sub-MIC without significantly compromising bacterial viability.

GC–MS analysis revealed that TTC is rich in various chromone derivatives (Table 2). To investigate the key chemicals in agarwood essential oil’s QSI activity, we selected two major components identified in GC–MS analysis for preliminary QS inhibitory screenings (Figure 4), which showed that 2-phenethyl-4H-chromen-4-one exhibited moderate QSI activity. However, its potency remained significantly weaker than that of the agarwood essential oil (TTC). When the two major volatile components were combined in proportions matching their relative abundance in the essential oil, the observed inhibitory activity was higher than 2-phenethyl-4H-chromen-4-one alone, yet still failed to achieve the inhibition level of the TTC essential oil. These findings suggest that agarwood essential oil may possess a multi-component synergistic mechanism: while individual compounds may show limited QS inhibitory activity, the combination can produce enhanced effects. However, the most QS inhibitory active of TTC essential oil likely depends on the concerted action of other minor components that were either untested or structurally complementary. This “synergistic enhancement” phenomenon provides scientific justification for the efficacy of using natural essential oils in their entirety. It also highlights the need for future research to investigate: (1) the potential roles of other components in agarwood oil, and (2) possible synergistic relationships between these constituents. The GC–MS results reflect only relative abundances of the detected components, and absolute quantification or compound isolation was not performed in this study. Further quantitative validation will be required to determine the precise contribution of individual constituents.

Virulence factors, as critical mediators enabling bacterial colonization, host tissue destruction, and immune evasion, are often precisely regulated by quorum sensing systems (Li et al., 2022). The pathogenicity of *S. aureus* is closely associated with its secretion of multiple hemolysins. Based on antigenic characteristics, these toxins can be classified into *α*, *β*, *γ*, and *δ* subtypes. Among them, α-hemolysin (Hla) is particularly crucial due to its potent lytic activity against host cells. Studies demonstrate that nearly 90% of clinically isolated pathogenic strains secrete this exotoxin (Tkaczyk et al., 2013). The results of this study reveal that at sub-MICs (125 μg/mL and 62.5 μg/mL), TTC could remarkably reduce hemolytic activity (Figure 7). This discovery identifies a promising candidate molecule for developing novel anti-virulence agents. By specifically neutralizing Hla pathogenicity rather than directly killing pathogens, TTC may present a lower risk of inducing resistance. Future mechanistic research will be carried out through two pathways after treatment with TTC: (1) downregulation of α-hemolysin gene expression to reduce Hla secretion, and (2) direct binding to extracellular Hla protein, interfering with its oligomerization process and consequently blocking its erythrocyte-lytic activity.

In addition to biofilm formation and hemolytic activity, TTC also significantly downregulates the expression of several genes related to biofilm formation, adhesion, and quorum sensing, including *icaA*, *icaD*, *clfA*, *clfB*, and *agrA* (Figure 8). Genes *icaA* and *icaD* in *S. aureus* could synergistically promote the synthesis of polysaccharide intercellular adhesin (PIA), enhancing its biofilm-forming capacity on food-contact surfaces (Chen et al., 2016). Meanwhile, *clfA* and *clfB* encode clumping factor proteins that mediate bacterial adhesion to host proteins or substrates, facilitating colonization (Chen et al., 2016). Upon sensing the extracellular autoinducing peptide (AIP) signal, agrA becomes phosphorylated and activates the P2 and P3 promoters, thereby upregulating the expression of *RNAII* and *RNAIII*, which in turn regulate the transcription of multiple virulence factors. Through this pathway, *agrA* participates in the transition of the bacterium from an adhesive to an aggressive virulent phenotype, playing a critical role in toxin secretion, biofilm dispersal, and immune evasion (Inagaki et al., 2024). The expression of these virulence factors is closely associated with QS systems and may influence bacterial survival and pathogenicity in food-processing environments. Notably, *icaA*, a key gene in biofilm formation of *S. aureus*, and *agrA*, one of the regulatory genes in QS, are both significantly downregulated. The downregulation of these genes may reduce the attachment ability of *S. aureus* to high protein foods such as meat and dairy products (Zhang et al., 2021). TTC is considered to have the potential to interfere with the quorum sensing (QS) system, possibly by disrupting the signal transduction process regulated by QS between bacteria, thereby reducing the activity of QS-related genes. Although the specific inhibitory mechanism still requires further investigation, this process ultimately weakens the ability of bacteria to form biofilms and makes the cells more likely to remain in a planktonic state. It should be noted that the downregulation of QS-related genes observed in this study provides indirect evidence of quorum-sensing interference rather than a direct confirmation of signal disruption. Since qRT-PCR reflects transcriptional responses at the cellular level, additional assays such as signal molecule quantification or QS reporter-based systems would be required to verify whether TTC directly interferes with autoinducing peptide–mediated communication. Therefore, the current findings should be interpreted as indicating a potential anti-QS effect, which warrants further mechanistic validation in future work.

Molecular docking results revealed that the representative compound 2-phenethyl-4H-chromen-4-one exhibited strong binding affinity with the biofilm-associated key protein IcaA, with a minimum binding energy of −8.4 kcal/mol (Figure 9). In contrast, its binding affinity with the quorum sensing (QS)-related protein AgrA was relatively weak (Supplementary Figure S4). This observation is inconsistent with the qPCR results. Such discrepancies may be attributed to the inherent limitations of molecular docking, which is based on static structural models and fails to capture the complexity of intracellular regulatory networks. Moreover, the multiple components present in TTC may act synergistically or influence upstream regulatory elements of the QS system, thereby indirectly enhancing the suppression of AgrA expression. Given the central regulatory role of AgrA in the QS system of *S. aureus*, even minor perturbations in its expression could trigger significant downstream transcriptional responses. Molecular docking primarily reflects the binding affinity between ligands and target proteins; however, it cannot fully explain the biological activity of a compound. TTC may exert its quorum sensing inhibitory effects through alternative mechanisms that are not captured by the docking model. Therefore, despite the relatively low binding energy, TTC may still possess potential as a quorum sensing inhibitor (QSI), and its biological efficacy requires further experimental validation.

In the molecular docking analysis, thymol (32.7%), the major component of the previously reported Lippia origanoides essential oil (LOTC II), was selected as a positive control. LOTC II has been reported to inhibit approximately 70% of *S. aureus* biofilm formation at 0.40 mg/mL, accompanied by reduced transcription of several biofilm- and quorum sensing–related genes, including *agrA*, *icaA*, and *hla* (Martínez et al., 2023). The docking results for thymol showed binding scores of −6.8 kcal/mol for *clfA*, −5.8 kcal/mol for *clfB*, −6.3 kcal/mol for *icaA*, −5.5 kcal/mol for *icaD*, and −5.3 kcal/mol for *agrA*, which were weaker than those observed for the representative TTC constituent 2-phenethyl-4H-chromen-4-one. It is important to note that molecular docking primarily reflects the theoretical binding affinity between ligands and target proteins, and cannot fully predict biological activity in living systems. When comparing LOTC II and TTC at the whole–essential oil level, TTC achieved a comparable or stronger biofilm inhibition rate (~78%) at a much lower concentration (125 μg/mL, 1/4 MIC), without affecting bacterial growth. TTC also downregulated genes associated with adhesion, biofilm regulation, and quorum sensing, and reduced α-hemolysin activity. These findings suggest that, although both essential oils exhibit anti-biofilm and anti-virulence properties, TTC may exert its effects at lower active concentrations while maintaining selectivity toward virulence suppression rather than bacterial growth inhibition. A direct head-to-head comparison under identical experimental conditions would be required to clarify the relative efficacy of the two essential oils. For this reason, LOTC II represents a suitable positive control candidate for future validation experiments.

In summary, this study not only confirms the QSI activity of agarwood essential oil but also provides scientific evidence for novel applications of natural plant resources in food preservation and pathogenic bacteria control. As an essential oil derived from traditional aromatic plants, agarwood possesses favorable flavor characteristics and potential safety advantages. This makes it promising as a green, safe, and low-risk food protection agent for applications such as food contact surface treatment, active packaging development, or antimicrobial additive formulation. Future research should further investigate its antimicrobial stability in actual food systems, safety profile, and strategies for the extraction and purification of key active components to support its industrial-scale applications.

## 5 Conclusion

This study systematically evaluated the quorum sensing inhibitory activity of four different types of *Aquilaria sinensis* essential oils against *S. aureus*. The results showed that the supercritical CO₂-extracted Tong-Ti-Xiang type essential oil (TTC) significantly inhibited biofilm formation and α-hemolysin activity at sub-inhibitory concentrations without affecting bacterial growth. TTC also downregulated several key genes related to biofilm formation, adhesion, and quorum sensing, including *icaA*, *clfA*, and *agrA*. GC–MS and molecular docking analyses suggested that TTC is rich in various chromone derivatives, which may contribute to its quorum sensing inhibitory effects. Overall, these findings indicate that TTC has promising anti-virulence and anti-biofilm potential, warranting further investigation to assess its stability, safety, and practical applicability as a natural antimicrobial agent in food systems.

## Data Availability

The original contributions presented in the study are included in the article/Supplementary material, further inquiries can be directed to the corresponding authors.

## Footnotes

1
https://www.uniprot.org/

2
https://pubchem.ncbi.nlm.nih.gov/
