# Supplementary material for: Aquilaria sinensis essential oil inhibits biofilm formation and virulence of Staphylococcus aureus
Source: Front Microbiol. 2025 Dec 1;16:1697407. doi: 10.3389/fmicb.2025.1697407 (PMC12702913; doi:10.3389/fmicb.2025.1697407)
Supplement: Supplementary file 1 [file Presentation_1.pdf]

Supplementary materials

Aquilaria sinensis Essential Oil Inhibits Biofilm Formation and Virulence of *Staphylococcus aureus*

Fang Wang[1#], Zhi-Wen Ding[1#], Ying-Jie Wang[1], Kai-Zhong Xu[1], Owias Iqbal Dar1[3], Min Wang[4] ,Sivasamy Sethupathy[4]Yangyang Liu[2*], Shi Tang[1*]

[1]Key Laboratory of Tropical Biological Resources of Ministry of Education, School of Pharmaceutical Sciences, Hainan University, Haikou 570228, China; fangwang0305@163.com (Fang Wang); dingzhiwen070@163.com (Zhi-Wen Ding); wyingjie2022@163.com (Ying-jie Wang); xu-kaizhong@foxmail.com (Kai-zhong Xu); tangshi705@hainanu.edu.cn (Shi Tang)

[2]Hainan Provincial Key Laboratory of Resources Conservation and Development of Southern Medicine & International Joint Research Center for Quality of Traditional Chinese Medicine, Hainan Branch of the Institute of Medicinal Plant Development, Chinese Academy of Medical Sciences and Peking Union Medical College, Haikou 570311, China; yyliu@implad.ac.cn (Yangyang Liu)

[3]Key Laboratory of Ministry of Education for Advanced Materials in Tropical Island Resources, Department of Chemistry and Chemical Engineering, Hainan University, Haikou 570228, China; owiaszolo@gmail.com (Owias Iqbal Dar)

[4]Department of Pharmacy, Hainan General Hospital (Hainan Affiliated Hospital of Hainan Medical University), Hainan, China, wangmin2020@muhn.edu.cn (Min Wang)

*Correspondence: Shi Tang, Key Laboratory of Tropical Biological Resources of Ministry of Education, School of Pharmaceutical Sciences, Hainan University, Haikou 570228, Hainan, China, E-mail: tangshi705@hainanu.edu.cn.

Yangyang Liu, Hainan Provincial Key Laboratory of Resources Conservation and Development of Southern Medicine & International Joint Research Center for Quality of Traditional Chinese Medicine, Hainan Branch of the Institute of Medicinal Plant Development, Chinese Academy of Medical Sciences and Peking Union Medical College, Haikou 570311, China, E-mail: yyliu@implad.ac.cn.

[#]These authors contributed equally to this work.

Table S1. The extract methods of four *A. sinensis* essential oil samples

essential oil	extraction method	source material
TTC	supercritical CO_2 extraction	Tong-Ti-Xiang
TTS	steam distillation	
HLC	supercritical CO_2 extraction	Huo-Lao-Xiang
HLS	steam distillation	

Table S2. Primers used for RT-qPCR

Genes	Primers	Sequences (5' to 3')
icaA	*icaA-F*	CTTGCTGGCGCAGTCAATAC
	icaA-R	CCAACATCCAACACATGGCA
icaD	*icaD-F*	CGCTATATCGTGTGTCTTTTGGA
	icaD-R	TCGCGAAAATGCCCATAGTT
clfA	*clfA-F*	TTACGAATCAGTTGACGAATGTG
	clfA-R	AGGCACTGAAAAACCATAATTCA
clfB	*clfB-F*	TGCAAGTGCAGATTCCGAAAAAAAC
	clfB-R	CCGTCGGTTGAGGTGTTTCATTTG
agrA	*agrA-F*	ACGTGGCAGTAATTCAGTGTATGTT
	agrA-R	GGCAATGAGTCTGTGAGATTTTGT
16S rRNA	*16S rRNA-F*	AGCCGACCTGAGAGGGTGA
	16S rRNA-R	TCTGGACCGTGTCTCAGTTCC

Primers were designed using the Primer-Blast module of the online website NCBI (https://www.ncbi.nlm.nih.gov/).

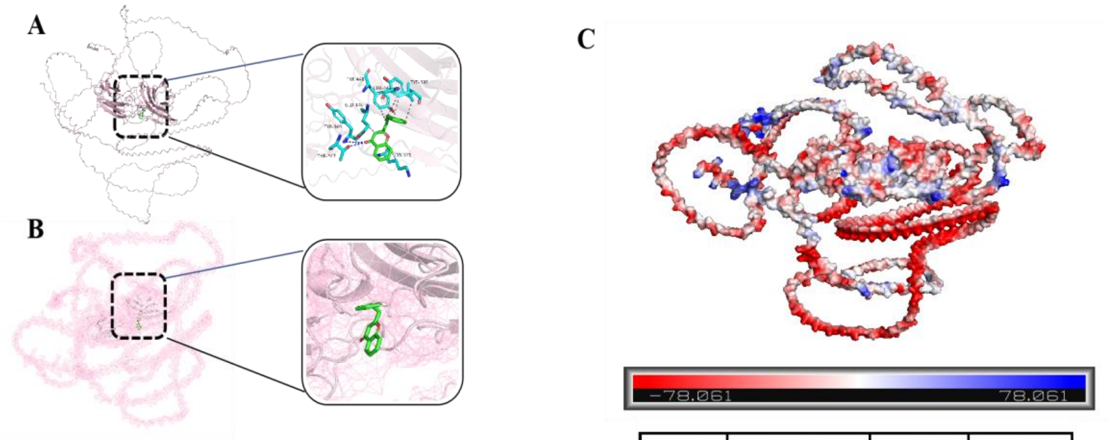

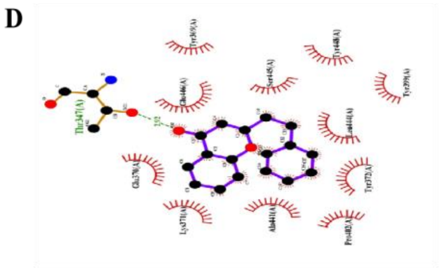

E

mode	Affinity（kcal/mol）	rmsd l.b	rmsd u.b
1	-8.4	0.000	0.000
2	-7.8	2.082	3.240
3	-7.3	2.727	4.715
4	-6.7	40.924	42.962
5	-6.7	3.562	5.725

Fig. S1.Molecular docking of 2-phenethyl-4H-chromen-4-one with the QS receptor protein *clfA*. (A) Three-dimensional structure of the 2-phenethyl-4H-chromen-4-one-clfA molecular docking. (B) 2-phenethyl-4H-chromen-4-one located within the molecular pocket of the *clfA* protein. (C) Surface electrostatic forces of the 2-phenethyl-4H-chromen-4-one-*clfA* complex. (D) Two-dimensional structure of the 2-phenethyl-4H-chromen-4-one-*clfA* interaction. (E) Lowest binding energy pose of the 2-phenethyl-4H-chromen-4-one-*clfA* molecular docking.

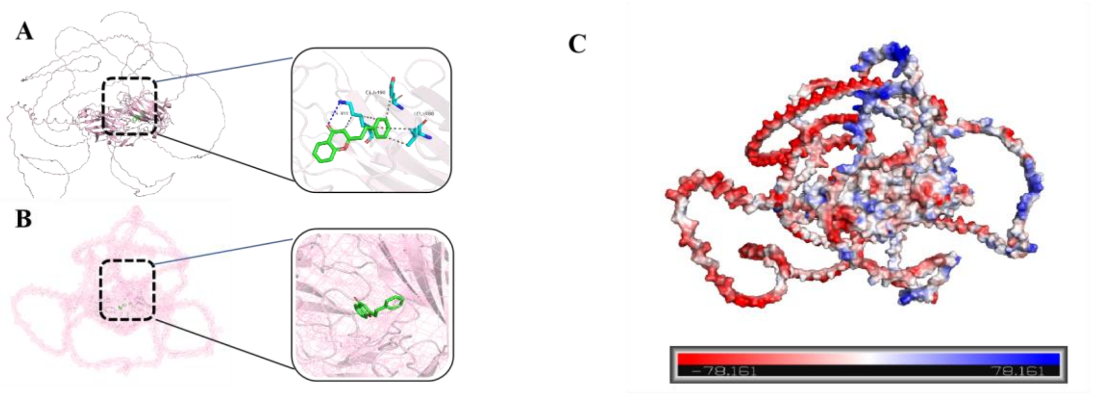

D

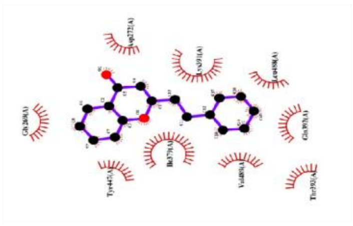

E

mode	Affinity （kcal/mol）	rmsd l.b	rmsd u.b
1	-7.9	0.000	0.000
2	-7.6	16.643	19.152
3	-7.4	1.865	2.935
4	-7.3	1.283	1.965
5	-7.3	2.217	7.413

Fig. S2.Molecular docking of 2-phenethyl-4H-chromen-4-one with the QS receptor protein *clfB*. (A) Three-dimensional structure of the 2-phenethyl-4H-chromen-4-one-*clfB* molecular docking. (B) 2-phenethyl-4H-chromen-4-one located within the molecular pocket of the *clfB* protein. (C) Surface electrostatic forces of the 2-phenethyl-4H-chromen-4-one-*clfB* complex. (D) Two-dimensional structure of the 2-phenethyl-4H-chromen-4-one-*clfB* interaction. (E) Lowest binding energy pose of the 2-phenethyl-4H-chromen-4-one-*clfB* molecular docking.

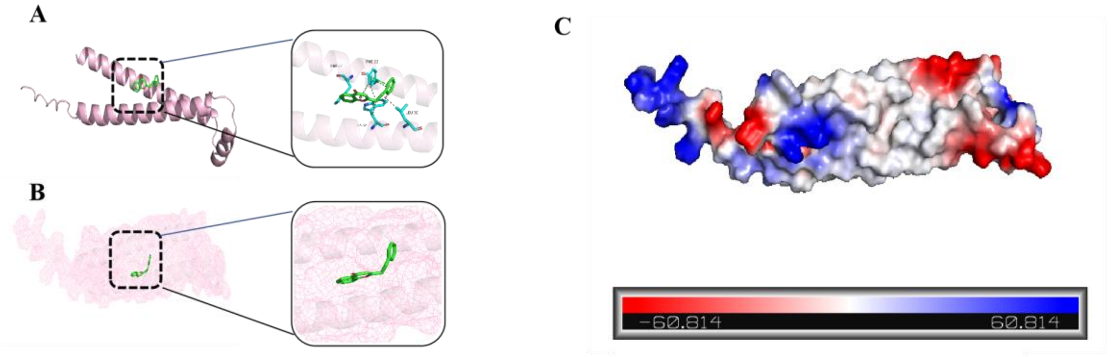

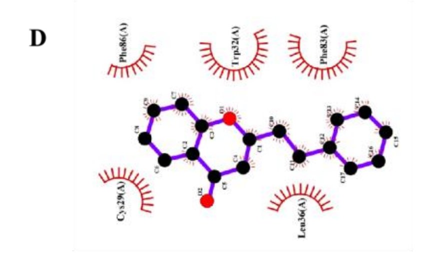

E

mode	Affinity（kcal/mol）	rmsd l.b	rmsd u.b
1	-6.9	0.000	0.000
2	-6.8	23.834	26.192
3	-6.7	15.292	19.363
4	-6.7	15.469	19.461
5	-6.6	7.874	12.948

Fig. S3.Molecular docking of 2-phenethyl-4H-chromen-4-one with the QS receptor protein *icaD*. (A) Three-dimensional structure of the 2-phenethyl-4H-chromen-4-one-*icaD* molecular docking. (B) 2-phenethyl-4H-chromen-4-one located within the molecular pocket of the *icaD* protein. (C) Surface electrostatic forces of the 2-phenethyl-4H-chromen-4-one-*icaD* complex. (D) Two-dimensional structure of the 2-phenethyl-4H-chromen-4-one-*icaD* interaction. (E) Lowest binding energy pose of the 2-phenethyl-4H-chromen-4-one-*icaD* molecular docking.

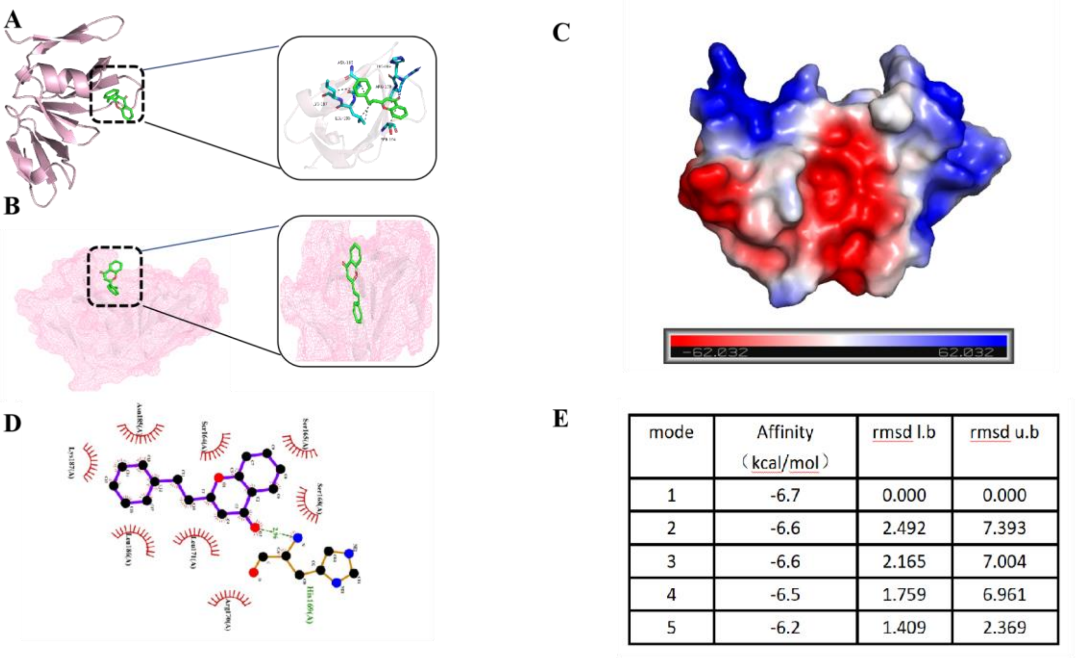

mode	Affinity （kcal/mol）	rmsd l.b	rmsd u.b
1	-6.7	0.000	0.000
2	-6.6	2.492	7.393
3	-6.6	2.165	7.004
4	-6.5	1.759	6.961
5	-6.2	1.409	2.369

Fig. S4Molecular docking of 2-phenethyl-4H-chromen-4-one with the QS receptor protein *agrA*. (A) Three-dimensional structure of the 2-phenethyl-4H-chromen-4-one-*agrA* molecular docking. (B) 2-phenethyl-4H-chromen-4-one located within the molecular pocket of the *agrA* protein. (C) Surface electrostatic forces of the 2-phenethyl-4H-chromen-4-one-*agrA* complex. (D) Two-dimensional structure of the 2-phenethyl-4H-chromen-4-one-*agrA* interaction. (E) Lowest binding energy pose of the 2-phenethyl-4H-chromen-4-one-*agrA* molecular docking.

Abbreviations

S.aureus Staphylococcus aureus

QS quorum sensing

QSI quorum sensing inhibitor

MIC the minimum inhibitory concentration

TSB tryptic soy broth

DMSO dimethyl sulfoxide

PBS phosphate buffered saline

CLSM confocal laser scanning microscopy

RT-qPCR quantitative real-time polymerase chain reactions
